# How to restore rotation center in total hip arthroplasty for developmental dysplasia of the hip by recognizing the pathomorphology of acetabulum and Harris fossa?

**DOI:** 10.1186/s13018-019-1373-9

**Published:** 2019-10-29

**Authors:** Heng Zhang, Jiansheng Zhou, Jianzhong Guan, Hai Ding, Zhiyan Wang, Qirong Dong

**Affiliations:** 1 Department of Orthopedics, The First Affiliated Hospital of Bengbu Medical College, Laboratory of Tissue and Transplant in Anhui Province, Bengbu Medical College, Bengbu City, Anhui Province China; 2 Department of Orthopedics, The Second Affiliated Hospital of Soochow University, Suzhou City, Jiangsu Province China

**Keywords:** Hip joint, Harris fossa, Rotation center, Arthroplasty, Hip dysplasia

## Abstract

**Purpose:**

To restore rotation center exactly in total hip arthroplasty (THA) is technically challenging for patients with end-stage osteoarthritis due to developmental dysplasia of the hip (DDH). The technical difficulty is attributable to the complex acetabular changes. In this study, we investigated the pathomorphology of acetabulum and Harris fossa of Crowe types I to IV and discussed the method of restoring rotation center of the hip.

**Methods:**

This study retrospectively reviewed 56 patients (59 hips) who underwent cementless THA due to end-stage osteoarthritis of DDH. The pathomorphology of acetabulum and Harris fossa was observed during operations. Using the preoperative and postoperative pelvic radiographs, the vertical and the horizontal distances of hip rotation center were measured in order to evaluate the effects of restoring rotation center of the hip.

**Results:**

Adult DDH acetabulum could be classified into four basic pathological types which include the shallow cup shape, the dish shape, the shell shape, and the triangular shape. Adult DDH Harris fossa could be classified into four pathological types, including the crack shape, the closed shape, the triangle shape, and the shallow shape, in accordance with the osteophyte coverage. The vertical and horizontal distances of hip rotation center on the pelvic radiographs before and after operations were as follows: the preoperative vertical distance of hip rotation center was (39.96 ± 5.65) mm, and the postoperative one was (13.83 ± 2.66) mm; the preoperative horizontal distance of hip rotation center was (42.15 ± 6.42) mm, and the postoperative one was (28.12 ± 4.56) mm.

**Conclusions:**

The acetabulum and Harris fossa can display different pathological types on account of different degrees of dislocation and osteophyte hyperplasia in the end-stage osteoarthritis of adult DDH. The hip rotation center can be accurately restored by locating the acetabular center with Harris fossa and acetabular notch as the marks.

Total hip arthroplasty remains the golden standard treatment to alleviate the pain and restore joint function of patients with end-stage osteoarthritis secondary to developmental dysplasia of the hip. It has been widely accepted that the acetabular prosthesis should be installed in the true socket as far as possible during the operation [1–6].

Several specialized surgical techniques have been developed to restore the rotation center in total hip arthroplasty (THA) [7–9]. However, these methods show the limitations to instruct orthopedist to restore the rotation center intraoperatively. In this study, it is reported that the pathomorphology of acetabulum and Harris fossa is observed through cases related with 56 adults who underwent THA. According to the anatomical mark of the Harris fossa and acetabular notch, acetabular prostheses are positioned exactly and the rotation center can be effectively restored.

## Materials and methods

This retrospective study was approved by the Ethics Committee on Human Research of the first affiliated hospital of Bengbu Medical College, and informed consent was obtained from all involved patients. The medical charts were assessed in terms of the criteria as below: hip end-stage osteoarthritis (OA Tonnis III stage) due to DDH (Crowe classification types I to IV), pain affected daily life and work, the muscle strength of the affected limb was normal, age range was from 30 to 65 years old, and no hip trauma history and other hip disease history. The exclusion criteria were hip osteoarthritis (OA Tonnis stages I to II), the hip joint pain was not obvious, the muscle strength of the affected limb was below IV class, ages older than 65 years old or younger than 30 years old, and hip trauma history or combined with other hip diseases. The study involved 56 patients who underwent primary cementless THA from March 2005 to March 2017. The 56 patients included 14 men (16 hips) and 42 women (43 hips). The average age is 54.14 ± 6.08 years (range from 30 to 65 years). The average body mass index is 23.51 ± 3.55 kg/m^2^ (range from 15.57 to 34.75 kg/m^2^). The average preoperative LLD was 2.84 ± 1.32 cm (range from 0.5 to 5.5 cm), in which 10 patients’ LLD is below 2 cm, 39 patients’ LLD between 2 and 5 cm, and 7 patients’ LLD above 5 cm. There were 8 hips of Crowe type I, 20 hips of Crowe type II, 18 hips of Crowe type III, and 13 hips of Crowe type IV. Patient demographics are shown in Table 1.

**Table 1:** Demographics of patients

Parameters	
Gender	
Male	14
Female	42
Age (years)	54.14 ± 6.08 (range, 30–65)
Body mass index (kg/m^2^)	23.51 ± 3.55 (range, 15.57–34.75)
LLD (cm)	2.84 ± 1.32 (range, 0.5–5.5)
Crowe classification	(three patients, bilateral DDH)
Crowe type I	8
Crowe type II	20
Crowe type III	18
Crowe type IV	13

The preoperative pelvic radiographs and three-dimensional CT scan were performed regularly for every case.

### Surgical technique

All cases were performed via a posterolateral approach in a lateral decubitus position. The femoral head, femoral neck, and proximal femur were exposed, and the sciatic nerve was carefully protected. The contracture soft tissue around the hip was released completely. Soft tissue and fat was cleared from the true acetabulum, which was identified by tracing and resecting the elongated and thickened joint capsule. The true acetabulum was exposed completely by placing three Hoffman retractors in the front, in the rear, and at the bottom of the acetabulum respectively. The morphological features of the acetabulum and Harris fossa were observed. In accordance with the degree of acetabular dysplasia and femoral head dislocation, the acetabulum presented four different types as follows: the shallow cup shape, the dish shape, the shell shape, and the triangular shape. According to the degree of osteophyte coverage in the bottom of the acetabulum, the Harris fossa presented four different types as below: the crack shape, the closed shape, the triangle shape, and the shallow shape. Although the anatomical pathomorphology of the acetabulum and Harris fossa presented the above trend in different Crowe types, there are also inter-sectional variations in our cases.

**Fig. 1:**
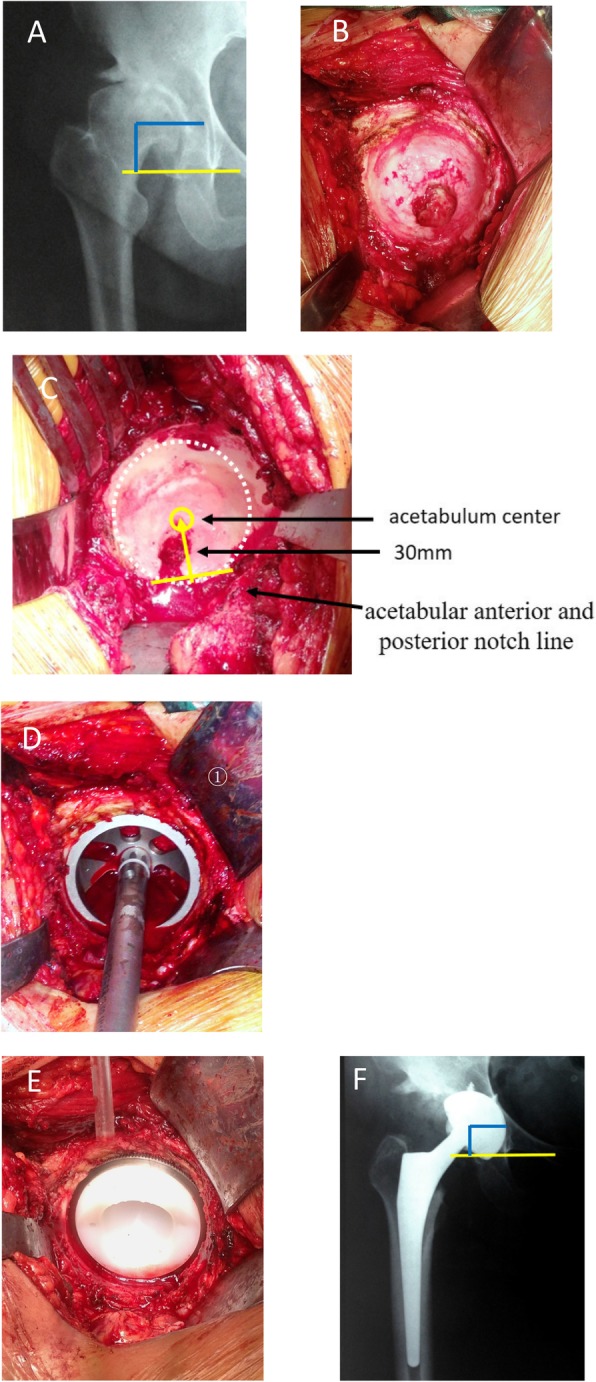
Radiographs of a 39-year-old female patient with end-stage osteoarthritis secondary to Crowe type I DDH. **a** Preoperative X-ray. **b** Intraoperative image showed shallow cup-shaped acetabulum and crack-shaped Harris fossa. **c** Locating the acetabulum center. **d** Reaming the acetabulum and installing the test cup. **e** Installing the acetabular prosthesis. **f** Postoperative X-ray and rotation center restoration

**Fig. 2:**
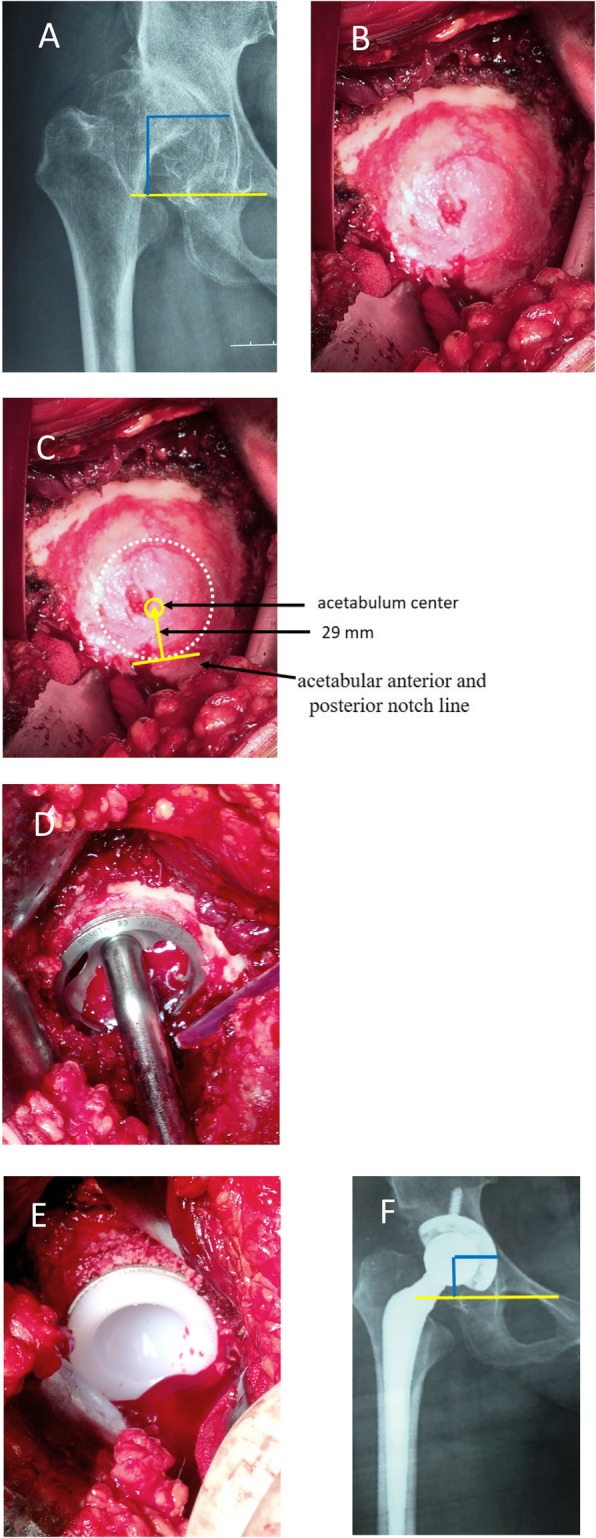
Radiographs of a 55-year-old male patient with end-stage osteoarthritis secondary to Crowe type II DDH. **a** Preoperative X-ray. **b** Intraoperative image showed dish-shaped acetabulum and closed shape Harris fossa. **c** Locating the acetabulum center. **d** Reaming the acetabulum and installing the test cup. **e** Installing the acetabular prosthesis. **f** Postoperative X-ray and rotation center restoration

**Fig. 3:**
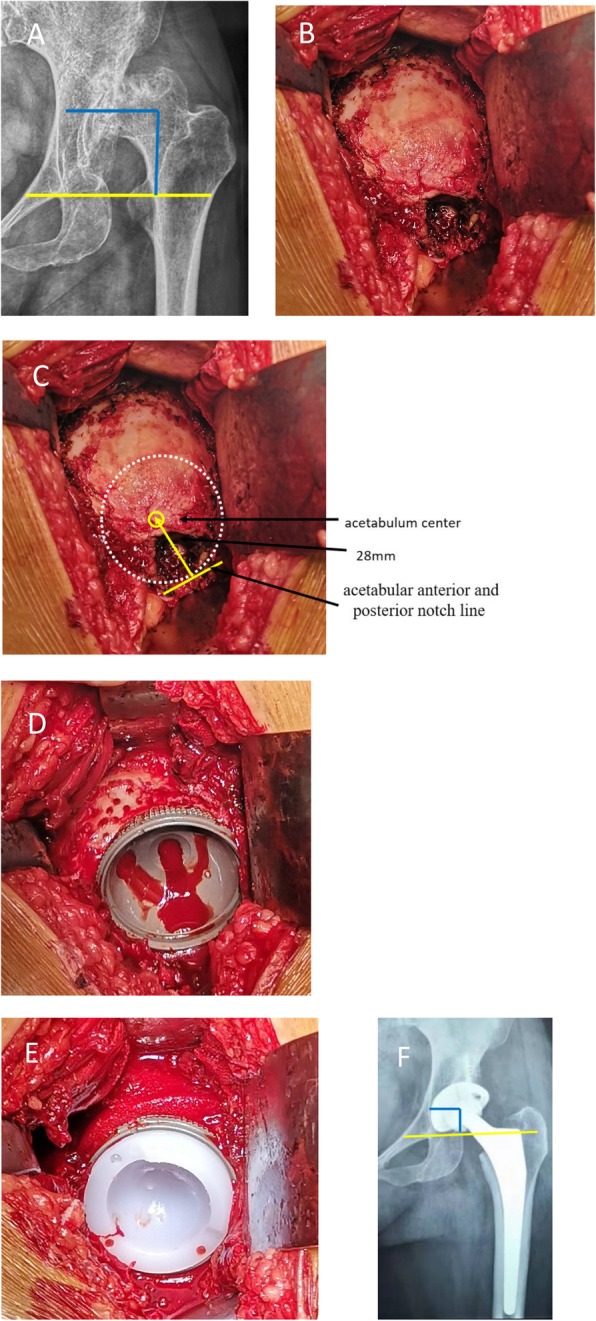
Radiographs of a 51-year-old female patient with end-stage osteoarthritis secondary to Crowe type III DDH. **a** Preoperative X-ray. **b** Intraoperative image showed the shell-shaped acetabulum and the triangle-shaped Harris fossa. **c** Locating the acetabulum center. **d** Reaming the acetabulum, installing the acetabular cup, and preparing the bone graft bed. **e** Installing the acetabular prosthesis and cancellous bone granule graft. **f** Postoperative X-ray and rotation center restoration

**Fig. 4:**
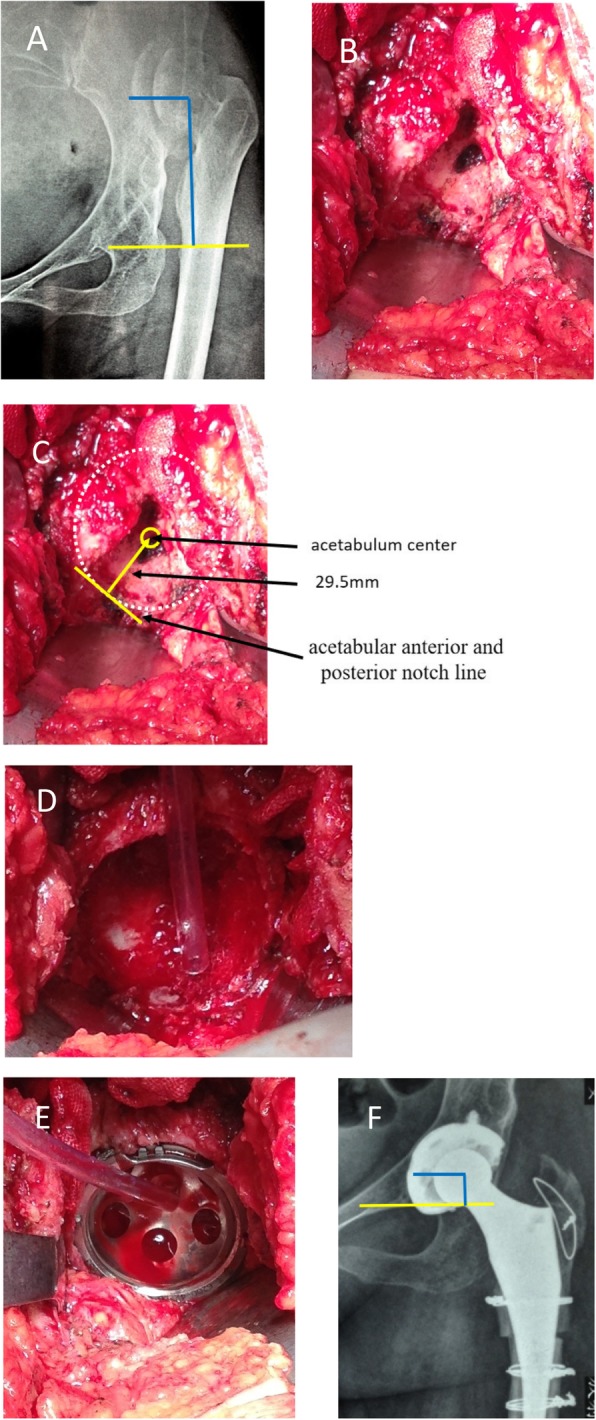
Radiographs of a 52-year-old female patient with end-stage osteoarthritis secondary to Crowe type IV DDH. **a** Preoperative X-ray. **b** Intraoperative image showed the triangular-shaped acetabulum and shallow-shaped Harris fossa. **c** Locating the acetabulum center. **d** Reaming the acetabulum. **e** Installing the acetabular cup. **f** Postoperative X-ray and rotation center restoration

Crowe type I acetabulum looked like a shallow cup, and its Harris fossa presented the crack shape (Fig. 1b). The crack happened on the bottom of the acetabulum and its width and depth varied in different cases. Using a bent probe (or small mosquito forceps) could find a potential space under the deep crack. The superficial layer of Harris fossa was covered by a lot of osteophytes. The Harris fossa could be recovered by removing the osteophytes to the edge of the interstitial space, which was filled with membrane-like tissues. Crowe type II acetabulum looked like a shallow dish, and its Harris fossa presented the closed shape (Fig. 2b). Although the Harris fossa was covered by thick osteophytes completely, there was still a potential space under the osteophyte. The Harris fossa could be recovered by removing the osteophytes to the edge of the interstitial space, which was also filled with membrane-like tissues. Crowe type III acetabulum showed the shell shape, and its Harris fossa presented the triangular shape (Fig. 3b). Using a bent probe could find a potential space above the triangle, which was also covered by thick osteophytes and filled with membrane-like tissues. The Harris fossa could be recovered by removing the osteophytes to the edge of the interstitial space. Crowe type IV acetabulum showed the triangle type, while its Harris fossa presented a flat triangle which was covered by few osteophytes (Fig. 4b). The Harris fossa could be recognized easily during the operation.

According to Jiansheng zhou’s acetabular center localization method, the acetabular center was located at 25~31 mm (mean 28 mm, depending on the size of the acetabulum) above the perpendicular bisector of the acetabular anterior and posterior notch line after the Harris fossa was recovered [10]. The acetabular center was also at the cephalic side of the Harris fossa, near the semilunar cartilage. The acetabular socket was reamed at 15° ± 10° of anteversion and 40° ± 10° of inclination from small to large, aiming at the acetabular center. Reaming was at concentric circles of the acetabular center and to the depth of Harris fossa’s bottom, paying much attention to the anterior and posterior wall of the acetabulum during reaming, especially the thickness of the anterior wall. The final size of the grind acetabulum was determined according to the criterion that the anterior and posterior wall had sufficient bone to maintain the stability of the cup prosthesis. If the acetabular component surface was not in contact with viable host bone well, bone wax was used to measure bone defect. If bone defect < 10%, bone graft was not needed. If bone defect > 10%, cancellous bone granules from the trimmed femoral head autograft were performed to provide adequate superolateral coverage of the cup. We inserted the cementless acetabular cup by press-fit fixation alone, although additional screws were applied to reinforce the initial stability of the cup if required.

### Statistical analysis

All statistical analyses were performed by using SPSS software for Windows (version 19.0; SPSS, Chicago, IL). Continuous variables were presented as means and ranges and categorical variables as frequencies. The two-sided paired *T* test was used for comparison between preoperative and postoperative hip rotation center data measurement on pelvis radiographs. Non-parametric methods Kruskal-Wallis *H* test was used for comparison among pathological types of acetabulum, Harris fossa, and Crowe types. A *P* value < .05 was considered statistically significant.

## Results

In this study, the 59 hips presented four pathological types of acetabulum respectively as follows: the shallow cup shape, the dish shape, the shell shape, and the triangular shape, while Harris fossa was classified into four pathological types correspondingly, including the crack shape, the closed shape, the triangle shape, and the shallow shape. The shell shape type of the acetabulum was further divided into three sub-types, the shell shape, the inverse shell shape, and the positive shell shape. The four pathomorphological types of acetabulum and Harris fossa correspond to Crowe types (Hc = 45.52, *P* < 0.01) (Table 2).

**Table 2:** The correlation between pathomorphological types of acetabulum, Harris fossa, and Crowe types

	Crowe types	I	II	III	IV
Harris fossa type	Crack shape	7	1	1	
	Closed shape	1	17	2	
	Triangle shape		2	15	2
	Shallow shape				11
Acetabulum type	Shallow cup shape	6	1		1
	Dish shape	2	18	1	
	Shell shape		1	17	
	Triangle shape				12

The acetabulum was reamed, and the acetabular test cup was hit into the socket by using the unified method; however, the site and size of acetabular bone defect were different, which affected the initial stability of the acetabular prosthesis. The bone bed of the reamed shallow cup shape (often occurred in Crowe type I) acetabulum could well accommodate to the cup (Fig. 1d). Bone graft was generally not required, and good initial stability could be obtained (Fig. 1e). The bone defect of the reamed dish-shaped acetabulum (often occurred in Crowe type II) was often located at the superolateral part of the acetabulum and was crescent-shape (Fig. 2d). The defect range was often less than 30%, which did not affect initial stability. Bone mud or cancellous femoral head could be used for granule bone graft (Fig. 2e). The bone defect of the reamed shell-shaped acetabulum (often occurred in Crowe type III) was often located at the anteriorosuperolateral and superolateral part of the acetabulum, sometimes at the posteriosuperolateral part (Fig. 3d). The bone defect was sickle-shape (Fig. 3d). The defect range was often > 30%. The initial stability was deficient. Impacting granule bone graft technique was used to obtain good cup coverage and stability (Fig. 3e). The triangular acetabulum was found only in Crowe IV (Fig. 4b). Although the bone mass was limited, good inclusivity and stability could be obtained by using a small acetabular cup (38–42 mm size). Bone graft was generally not required (Fig. 4e). The proportion of acetabular defects were correlated to acetabular pathomorphology.

The acetabular center was located, and the acetabular prosthesis was installed by using Harris fossa and acetabular notch as the marks. The vertical and horizontal distances of hip rotation center on the pelvic radiographs before and after surgery were as follows: the preoperative vertical distance of hip rotation center was (37.96 ± 5.65) mm, and the postoperative one was (13.83 ± 2.66) mm; the preoperative horizontal distance of the hip rotation center was (42.15 ± 6.42) mm, and the postoperative one was (25.12 ± 4.56) mm. The difference was statistically significant (*t* = 3.768, *P* < .05).

## Discussion

Performing primary THA for a patient with DDH remains a challenge, because of changed acetabular shape, position, and orientation caused by developmental dysplasia [11–13]. Many scholars advocate that acetabular prosthesis should be installed in the true acetabulum [1–6]; however, what are the pathological types and characteristics of DDH acetabulum and how to localize the center of the acetabulum and restore the rotation center of the hip are still controversial and lack of deep study. Based on the fundamental research of the correlation between the center of the acetabulum and the Harris fossa, the acetabular notch [10], the pathomorphology, and the anatomical character of DDH acetabulum and Harris fossa, how to recover the fossa from the pathological states and how to restore the rotation center were further discussed in this paper.

Multerer et al. [14] reported that DDH was caused by a variety of anatomical abnormalities, including muscles around the hip, the acetabulum, and the femur. The pathomorphology of DDH acetabulum was related to many factors. We agree with this viewpoint. Based on our research, the pathomorphology of DDH acetabulum is related not only to developmental dysplasia, but also to the degree of hip dislocation. The pathomorphology of Harris fossa is mainly related to the degree of osteophyte hyperplasia. Because the acetabulum has not suffered friction and stress caused by femoral head weight since birth, Crowe type IV acetabulum basically maintains a small and shallow triangular dysplasia state [15, 16]. Harris fossa also presents a mostly flat triangle. The anterior and posterior notch of the Harris fossa are easy to be recognized. Compared with other types, the acetabulum of DDH Crowe type I contacts with the femoral head at most, so the acetabulum presents to be a shallow cup and inferior osteophytes often cover the Harris fossa which often presents a crack shape. In Crowe type II, the contact and weight bearing position between the acetabulum and femoral head mainly occurs in the middle and upper part of the acetabulum which becomes precipitous, so the acetabulum presents to be a shallow dish shape. Due to lots of osteophytes which are different in color from the normal bone, the Harris fossa presents a closed shape. In Crowe type III, the contact and weight bearing position between the acetabulum and femoral head mainly concentrated on the upper part of the acetabulum, because the effective load area decreases, the stress per unit area increases, and the acetabulum presents the shell shape with a broad upper part and a narrow lower part [17]. The Harris fossa usually presents a triangular type which is covered by few osteophytes. In this study, the pathomorphology changes of the acetabulum and Harris fossa are found to relate with not only dysplasia development and femoral head dislocation degree, but also the courses of disease and living habits. So in each type of DDH, there will be the situation of cross occurrence of different pathological types. This is consistent with Macheras et al.’s views [18].

It was considered that the difference in the coverage of hyperplastic osteophytes over the Harris fossa was the pathological foundation for the different shapes of the adult DDH acetabulum and Harris fossa in our patient series. In some cases, the preoperative CT cross section could clearly reflect the different manifestations of osteophytes covering the Harris fossa and the Harris fossa space of osteophytes, which were consistent with the intraoperative observation results. Removing the surface osteophyte, the underlying Harris fossa can be visible intraoperatively. In Crowe type IV, the Harris fossa presents as the shallow shape, which is covered by few osteophytes and easy to identify. In the crack shape type and the triangle shape type, there is also an underlying space in the Harris fossa. The Harris fossa can also be visible by removing the osteophytes and the membrane structure. In the closed shape type, however, it is difficult to determine the exact position without experience, because the Harris fossa is covered completely by osteophytes. There are three methods for reference: (1) judging from the color—the osteophyte covering on the surface of Harris fossa is discolorated from the surrounding bone; (2) judging from the position—exposing the inferior part of the acetabulum, Harris fossa is located above the bottom, in the middle of the anterior and posterior wall of the acetabulum; and (3) judge by using a narrow bone chisel to the deep part of Harris fossa when you can feel empty. The bottom of the Harris fossa is a hard bone. The Harris fossa can be recovered by removing the osteophytes and the membrane tissues in the gap.

It has been reported in the previous studies that the center of the acetabulum was related to Harris fossa and acetabular notch in the normal hip joint. The center of the acetabulum was located at 25~31 mm, which the mean is 28 mm depending on the size of the acetabulum, above the perpendicular bisector of acetabular anterior and posterior notch line which was near to the articular cartilage surface on the cephalic side of Harris fossa [10]. We also used this method to determine the center of the acetabulum in DDH cases. The vertical and horizontal distances of the hip rotation center on the pelvic radiographs after surgery were as follows: the vertical distance of the hip rotation center was (13.83 ± 2.66) mm; the horizontal distance of the hip rotation center was (25.12 ± 4.56) mm. We found that the rotation center was moderately ingression. This was due to the flat true acetabulum of DDH and limited bone mass, especially in types II and III. We reamed the acetabular socket as deep as possible on the premise of bone integrity of the acetabular inner wall bone cortex in order to increase the coverage and the stability of the acetabular prosthesis. Proper ingression of the center of the hip joint is beneficial to the recovery of abduction strength of the hip joint [19]. Therefore, we suggest that the rotation center of the hip joint can be restored satisfactorily by recovering Harris fossa, locating the acetabular center, reaming the acetabulum at the concentric circle, and installing the acetabular prosthesis for the end-stage osteoarthritis of DDH cases.

Another important point of recovering Harris fossa in DDH total hip arthroplasty is beneficial to determine the reaming depth of the acetabulum. For a flat and steep DDH acetabulum, the reaming depth is often critical to coverage and initial stability of the acetabular prosthesis.

If reaming too shallowly, it will lead to deficient coverage of the prosthesis and deficient initial stability. If reaming too deeply, it is easy to break through the medial wall of the acetabulum, even causing central dislocation of the acetabular prosthesis [20]. In addition to the dysplasia abnormal anatomical structure, the acquired large number of osteophytes makes the acetabulum more flat, which often forms a “double-layer acetabulum” in DDH osteoarthritis. When the osteophytes were removed, not only can Harris fossa be reconstructed, but also the acetabular bottom can be found. Therefore, the hip rotation center can be accurately restored by locating the acetabular center with Harris fossa and acetabular notch as the marks. Reaming was at the concentric circle of the acetabular center and to the depth of Harris fossa’s bottom which can realize the coverage of the acetabular prosthesis and the clamping effect of the anterior and posterior acetabular walls as much as possible and also can avoid the phenomenon of wearing out at the same time. By using this method, in Crowe type I and IV cases, the acetabulum prosthesis could obtain good coverage and initial stability, the prosthesis coverage rate was > 90%, and bone graft was not necessary. However, in Crowe type II and III cases, the prosthesis coverage rate often was < 90%, and cancellous bone granules from the trimmed femoral head autograft were performed to provide adequate superolateral coverage of the cup and enhance initial stability.

Our study had several limitations. First, it was an independent study and the sample size was small. Second, there were several factors attributed to adult DDH acetabular morphology, including the course of the disease, living habits, occupations, and iatrogenic interventions. So, the pathomorphology of acetabulum and Harris fossa described in this study has its limitations which remained to be confirmed by more cases and studies.

## Conclusions

The acetabulum and Harris fossa can display different pathological types due to different degrees of dislocation and osteophyte hyperplasia in the end-stage osteoarthritis of adult DDH. The hip rotation center can be accurately restored by locating the acetabular center with Harris fossa and acetabular notch as the marks. This technique is simple, reproducible, manipulative, and effective to restore rotation center in total hip arthroplasty for developmental dysplasia of the hip.

## Data Availability

All data generated or analyzed during this study are included in this published article.

## Abbreviations

DDH: Developmental dysplasia of the hip
THA: Total hip arthroplasty
